# Associations between female sex hormones, estrous cycle, ischemic preconditioning and myocardial infarct size after ischemia–reperfusion injury

**DOI:** 10.1007/s00395-025-01099-9

**Published:** 2025-02-13

**Authors:** Tetiana Pylova, Ahmed Elmahdy, Maryna Krasnikova, Abhishek Jha, Erik Axel Andersson, Yalda Kakaei, Aaron Shekka Espinosa, Amin Al-Awar, Ermir Zulfaj, Amirali Nejat, Valentyna Sevastianova, Mana Kalani, Henrik Ryberg, Åsa Tivesten, Elmir Omerovic, Björn Redfors

**Affiliations:** 1 Department of Molecular and Clinical Medicine, Institute of Medicine, Sahlgrenska Academy, University of Gothenburg, Bla Straket 5 B Wallenberglab/SU, 413 45 Gothenburg, Sweden; 2 Wallenberg Centre for Molecular and Translational Medicine, Institute of Medicine, Sahlgrenska Academy, University of Gothenburg, Gothenburg, Sweden; 3 Department of Clinical Chemistry, Sahlgrenska University Hospital, Region Västra Götaland, Gothenburg, Sweden; 4 Department of Laboratory Medicine, Institute of Biomedicine, Sahlgrenska Academy, University of Gothenburg, Gothenburg, Sweden; 5 Department of Endocrinology, Sahlgrenska University Hospital, Region Västra Götaland, Gothenburg, Sweden; 6 Department of Cardiology, Sahlgrenska University Hospital, Region Västra Götaland, Gothenburg, Sweden

**Keywords:** Myocardial infarction, Ischemic preconditioning, Hormonal preconditioning, Ischemia–reperfusion injury

## Abstract

Studies on sex differences in myocardial infarction (MI) typically focus on males versus females, the exploration of hormonal physiologic variations and their impact on the infarct size remains limited. The objective of this study was to examine whether infarct size after myocardial ischemia/reperfusion injury in female rats differs in different phases of the estrous cycle, and according to the levels of sex hormones; and to assess whether the effect of ischemic preconditioning on infarct size varies in different phases of the estrous cycle and between sexes. Female rats were divided into three groups based on the estrous cycle: proestrus, estrus, and diestrus. A fourth group consisted of ovariectomized female rats. Male rats were included as a fifth group, and orchiectomized males as a sixth group. Each group underwent ischemia/reperfusion injury, with or without prior ischemic preconditioning (IPC). Plasma sex hormone levels were measured with gas chromatography-tandem mass spectrometry. Females in the proestrus showed significantly smaller infarct size compared to all other groups. Multivariable analyses identified proestrus, IPC, and estradiol as independent predictors of smaller infarct size while male sex and gonadectomy as independent predictors of larger infarct size. There was a statistical interaction between IPC and both sex and hormonal status, with a greater protective effect of IPC on infarct size in males and gonadectomized rats. After ischemia–reperfusion, proestrus female rats developed the smallest while male and gonadectomized rats the largest infarct size. Conversely, IPC conferred greater cardioprotection in male and gonadectomized rats than females in proestrus.

## Introduction

Myocardial infarction (MI) is a leading cause of death for both women and men. In recent years, increasing attention has been given to sex differences in the incidence and course of MI [4, 5, 15, 16]. While men has a higher incidence of MI, postmenopausal women face a significantly greater risk of complications, such as acute heart failure, and often have mortality rates comparable to or even higher than those of men [25, 37]. Historically, women have been under-represented in cardiovascular research, with studies, diagnostics, and treatments mainly based on the male physiology [25, 33, 39].

A significant distinction between sexes lies in the differences in sex hormones and regular hormonal cycles. There has been considerable research, including both preclinical experiments and clinical trials, aimed at understanding the impact of sex hormones on MI outcomes, primarily via pharmacologic interventions [18, 19, 26]. Female sex has been proposed to confer a protective effect against MI compared to male sex [5, 29, 30]. However, the extent to which sex hormones exert a protective or harmful effect remains a matter of debate [34, 36]. On the other hand, the impact of natural hormonal fluctuations (menstrual in women and estrous cycles in animals) on the development and prognosis of MI has been scarcely explored [27, 41]. Despite the limited research, the findings regarding the effects of these natural hormonal fluctuations on MI outcomes are contradictory, underscoring the necessity for more detailed investigation.

Among sex hormones, 17β-estradiol is known for its cardiovascular protective effects, mediated by three estrogen receptors present in the heart [17, 28]. These protective effects have been associated with ischemic preconditioning (IPC), i.e., brief episodes of ischemia that protect against subsequent prolonged ischemia and reduce infarct size [12, 31]. Interestingly, IPC and estrogen have been reported to share protective mechanisms [8, 40]. However, the variation in the effects of IPC across different phases of the hormonal cycle, during which estrogen fluctuates, has never been investigated. Furthermore, in previous animal studies on sex differences, the estrous cycle phases and the normal fluctuations in sex hormone levels were not taken into account, potentially leading to misinterpretation of results [20, 23]. Notably, the diestrus phase, characterized by low levels of estrogen, is the longest phase in the estrous cycle. Consequently, it is possible that a significant portion of the subjects in previous studies were in diestrus, impacting the generalizability of the findings. This must be accounted for when studies on sex differences are designed.

Levels of sex steroid hormones are commonly assessed using immunoassay-based techniques in both clinical and research environments. Nevertheless, the reliability of these assays, particularly regarding specificity, is often called into question, particularly when measuring hormones at lower concentrations [38]. Gas chromatography-tandem mass spectrometry (GC–MS/MS) represents the most advanced and reliable technique and provides a valuable tool to characterize the sex steroid metabolism in a variety of sex steroid-related rodent models and allow the analysis of sex steroid concentrations in samples with low hormone levels [32].

This study aimed to examine whether myocardial infarct size after myocardial ischemia–reperfusion in female rats differs in different phases of the estrous cycle, and according to the levels of sex hormones; and to assess whether the effect of ischemic preconditioning on infarct size is different in different phases of the estrous cycle and between sexes.

## Methods

### Animals

A total of 98 female and 46 male Sprague Dawley rats (body weight 250–350 g; aged eight to ten weeks Janvier Labs (Le Genest-Saint-Isle, France) were obtained and acclimatized at the Laboratory for Experimental Biomedicine (Gothenburg, Sweden) for 1 week before the start of the experiments. The rats were housed in a temperature-controlled (19–21 °C) facility with a 12-h light/dark cycle and had free access to food and water. All experiments were approved by the Gothenburg Animal Ethics Committee (Dnr 5.8.18–11014/2023) and performed after the ARRIVE guidelines and in accordance with the local legislation and institutional requirements.

### Study design

**Fig. 1:**
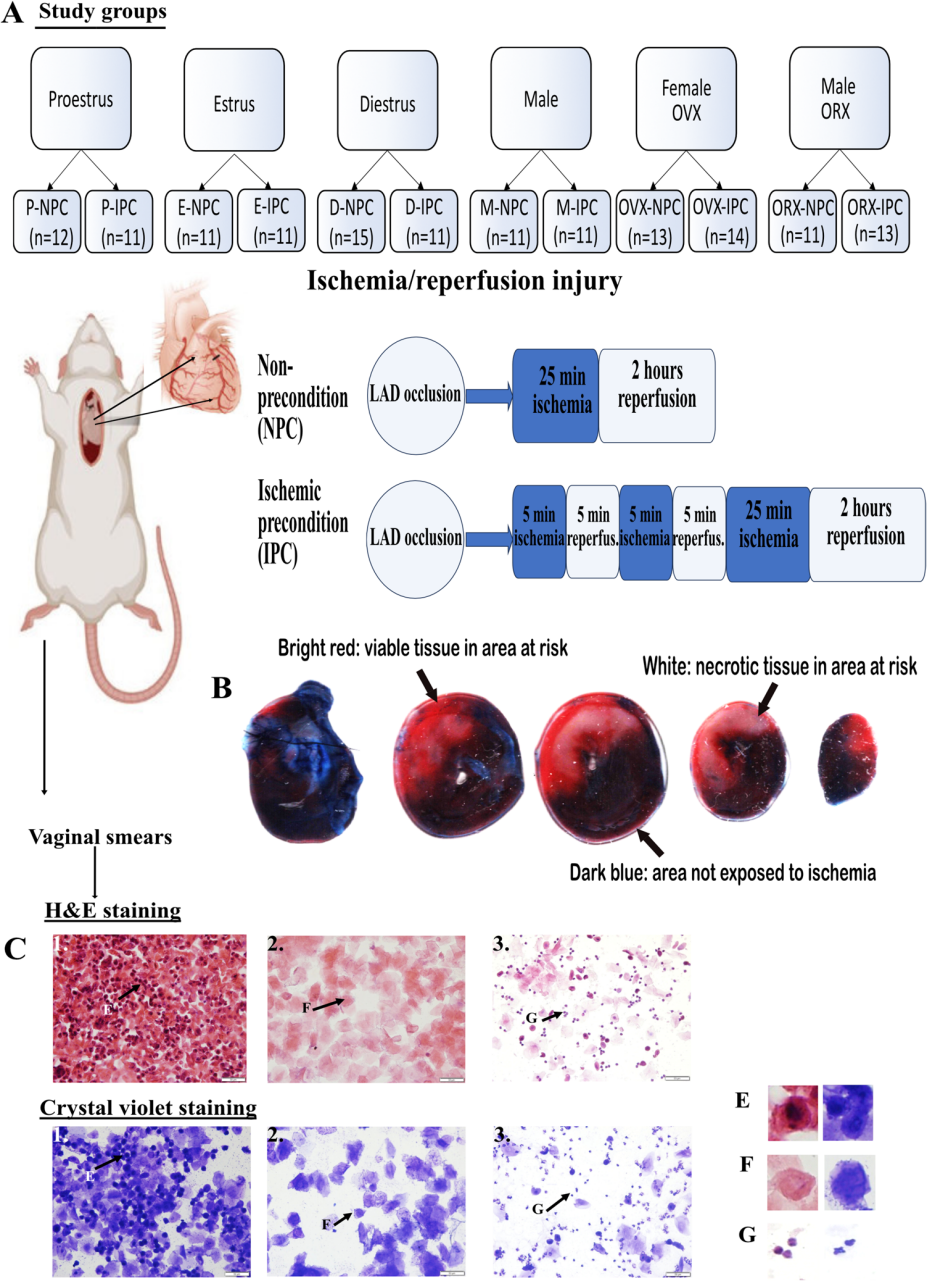
Study groups and the estrous cycle in female rats. (**A**) Female Sprague–Dawley rats were divided into groups based on the estrous cycle. Rats from all groups, including male, and castrated male and female rats, were exposed to ischemia/reperfusion injury with or without prior ischemic preconditioning. Infarct size was measured via Evan’s blue/triphenyltetrazolium chloride double staining. (**B)** Representative images of a whole heart stained by triphenyltetrazolium chloride. Dark blue areas represent myocardial regions not exposed to ischemia, while bright red and white areas represent the area at risk. Bright red indicates viable tissue, and white indicates necrotic tissue. (**C**) Representative vaginal smears using H&E and crystal violet staining for the four estrous cycle stages in female rats at 40 times magnification. 1. Proestrus characterized by nucleated epithelial cells (**E**). 2. Estrus characterized by large nonnucleated cornified epithelial cells (**F**). 3. Diestrus characterized by low cellularity, a combination of nucleated, nonnucleated cornified epithelial cells and neutrophils (**G**). Scale bar is 50 μm. P = proestrus; E = estrus; D = diestrus; M = male; H&E = hematoxylin–eosin; NPC = no ischemic preconditioning; IPC = ischemic preconditioning; ORX = orchiectomized rats; OVX = ovariectomized rats

All rats were followed 10–14 days before surgery to confirm sexual maturity and a regular 4-day cycle (Fig. 1). The study included six groups. Intact female rats (F) were divided into three groups according to their phase of the estrous cycle. These groups were (i) proestrus (P; *n* = 23), (ii) estrus (E; *n* = 22), and (iii) diestrus (D; *n* = 26). Rats in the metestrus phase were included in the diestrus group, as the metestrus phase is a transitional phase that lasts from 4 to 6 h with similar hormone levels and cytological smear patterns as the diestrus phase [14]. A fourth group consisted of ovariectomized female rats (OVX; *n* = 27). In addition to the female rats, male rats were included as a fifth group (M, *n* = 22) and orchiectomized males as a sixth group (ORX; *n* = 24). Within each of the six groups, rats were either exposed to ischemic preconditioning (IPC) or a sham procedure without ischemic preconditioning (NPC).

### Vaginal smears

The method for obtaining vaginal cell samples involved lavaging rat vaginas with saline using a pipette and transferring the collected material onto glass microscope slides. To ensure accurate analysis, a small amount of the collected sample is spread evenly across the slide surface. The slides were allowed to air-dry and then prepared for staining. All female rats were followed for 14 days before surgery to confirm sexual maturity and a regular 4-day cycle [24].

### Cytological staining using crystal violet

Crystal violet staining was used to determine the phase of the cycle for each rat daily, up to and including the day of surgery. Slides with smears were immersed in crystal violet stain for 1 min, followed by a 1-min wash in ddH_2_O. Excess water was removed from slide edges, and approximately 15 μl of Mounting medium (Pertex® Histolab, Gothenburg, Sweden) was pipetted onto the stained smear before it was mounted with a coverslip. This staining technique is a quick way of assessing the cellular composition of the vaginal smears, allowing for categorization of the rats into specific stages of the estrous cycle.

### Hematoxylin–eosin staining

After the surgeries, smears from all animals were subjected to hematoxylin–eosin staining as a gold standard to further confirm the stage of the estrous cycle. Staining were performed following a standard protocol [6].

### Vaginal cytology

Smears were examined on microscope to determine the present cell types. The cells lining the vagina of female rats corresponds to the levels of circulating hormones and provide a marker for the stage of cycle. The stages can be recognized by the presence, absence, or proportional numbers of epithelial nucleated cells, cornified (keratinized) cells, and leucocytes. The ratio of cells present is used to determine the estrous stage of the rat at the time of sample collection (Fig. 1). Crystal violet stains were examined with a light microscope (Kern, Frankfurt, Germany) and hematoxylin–eosin stains with an Olympus BX 60 Digital Microscope (Tokyo, Japan).

### Ovariectomy and Orchidectomy models

Female rats in fourth group underwent bilateral ovariectomy (OVX) at 7 weeks of age, as described previously [2]. Briefly, rats were anesthetized with isoflurane (Isoflutek®Vet 1000mg/g), a dorsal skin incision was made under anesthesia caudal to the posterior border of the ribs. The posterior abdominal muscle wall was bluntly dissected, the abdominal cavity was opened, and the ovary was gently exteriorized and removed. The uterine horn was returned into the abdomen. The muscles and skin incision were closed with sterile nylon sutures, and the process was repeated on the other side. After surgery, nonsteroidal anti-inflammatory drugs (Karprofen 50 mg/ml) were administered subcutaneously to all rats to reduce pain. Following recovery, rats were monitored for three weeks before undergoing heart surgery. To verify the model, blood samples were collected from the tails of OVX rats, and plasma concentrations of 17β-estradiol, progesterone, estrone, testosterone, and androstenedione were measured prior to heart surgery.

Male rats underwent bilateral castration (orchiectomy; ORX) under isoflurane anesthesia (Isoflutek®Vet 1000 mg/g), as described in a previous study [21]. The scrotum was opened and funiculus spermaticus was ligated with 4–0 polypropylene non-absorbable suture. The testes were removed, and the muscle layer and skin were closed with sutures. Subcutaneous injections were administered for postoperative analgesia. After recovery, male rats were also monitored for three weeks before heart surgery.

### Ischemia–reperfusion model

Ischemia–reperfusion with or without IPC was induced as previously reported [3, 10]. Briefly, rats were anesthetized with an intraperitoneal injection of Ketamine (100 mg/kg) and Xylazine (5 mg/kg) and intubated endotracheally and ventilated with a small-animal ventilator (SAR-1000: CWE Inc, PA, USA). To maintain surgical anesthesia a continuous infusion of ketamine and xylazine (0.125 mg/ml and 3 mg/ml, respectively) in Ringer solution was administered through cannulation of the lateral tail vein during the surgery. The levels of end-tidal CO2 were continuously monitored using a CapStar-100 End-Tidal CO2 monitor (CWE Inc) and kept between 5–6%. The left anterior descending artery (LAD) was temporarily ligated using a 6.0 suture (Ethicon Inc, NJ, USA). The LAD occlusions were confirmed by electrocardiogram changes and by observing cardiac akinesia on echocardiography. IPC was induced via two cycles of coronary artery occlusion for 5 min followed by 5 min of reperfusion before the main ischemic insult. Each group underwent 25 min ischemia and 2 h of reperfusion.

### Evan’s blue/triphenyltetrazolium chloride staining and infarct size determination

The myocardial infarct size was assessed by Evan’s blue/triphenyltetrazolium chloride (TTC) double staining as previously reported [1, 11]. After 2 h of reperfusion, the LAD was religated, and approximately 2 mL of 5% Evan’s blue dye was injected via the lateral tail vein to stain the nonischemic area [22]. Hearts were excised quickly; stored at − 20 °C overnight; then sliced transversely into five 2 mm thick slices from the apex to the base; and incubated in 1% TTC for 10 min at 37 °C in the dark. After incubation, tissue sections were fixed for 10 min in 10% formalin and then placed for 10 min in phosphate buffer (pH 7.4) for washing. Images were obtained with a scanner (Epson® Expression® Premium XP-7100, Japan), and ImageJ 1.34 (NIH, USA) was used to quantify the infarcted volume. The volume at risk and the infarcted volume were assessed in each section by two investigators who were blinded to the groups.

### Rat blood sampling and plasma collection analysis

Blood samples were obtained in lithium heparin–coated tubes via the tail vein in anesthetized animals between 7:30 am and 10:30 am before surgery. The samples were maintained at 4 °C before centrifuging at 2000x*g* for 10 min. After the centrifugation, plasma was collected and stored at − 80 °C until analysis.

### Gas chromatography-tandem mass spectrometry (GC–MS/MS)

Gas chromatography-tandem mass spectrometry (GC–MS/MS) was used to determine the sex steroid profile (estradiol, estrone, testosterone, progesterone, and androstenedione) in rat plasma as described in prior studies [32].

### Statistical analysis

All statistical analyses were conducted using IBM SPSS Statistics 27 and R version 4.2.0. All figures were generated using R, with additional formatting and design adjustments completed in Affinity Designer. Physiological parameters across different groups are presented as mean ± standard deviation (SD) and compared using a one-way ANOVA with Bonferroni correction for multiple comparisons. Infarct sizes and hormonal levels are expressed as median and interquartile range (IQR) and compared using the Kruskal–Wallis test followed by Dunn’s multiple comparisons test. Cohen’s Kappa assessed the agreement between crystal violet and hematoxylin–eosin staining techniques.

Multivariable linear regression was employed to evaluate independent associations and interaction effects across several models exploring factors influencing infarct size. The specific models applied were as follows:

*Sex and IPC* The first model examined the independent effects of sex and IPC on infarct size. Female sex and NPC served as reference groups, and an interaction term was included to assess whether IPC’s effect varied by sex.

*Gonadectomy and IPC* The second model investigated the relationship between OVX-females and ORX-males and IPC on infarct size. An interaction term was used to evaluate whether gonadectomy moderated the effect of IPC on infarct size.

*Sex, gonadectomy, and IPC* The third model included sex, gonadectomized groups, and IPC as independent variables. Interaction terms assessed whether IPC’s effects differed based on sex or gonadectomy.

*Estrous cycle phases and IPC* The fourth model investigated the effects of estrous cycle phases (proestrus, estrus, diestrus), male sex, and gonadectomy on infarct size, with proestrus and NPC as reference groups. An interaction term was incorporated to evaluate whether IPC’s effects varied across estrous phases.

*Hormonal levels and infarct size* The final model analyzed the association between hormonal levels and infarct size, with hormonal data log-transformed to address skewness. Estrone and androstenedione were excluded due to high variance inflation factor (VIF) values (> 30), indicating multicollinearity with estradiol.

## Results

**Fig. 2:**
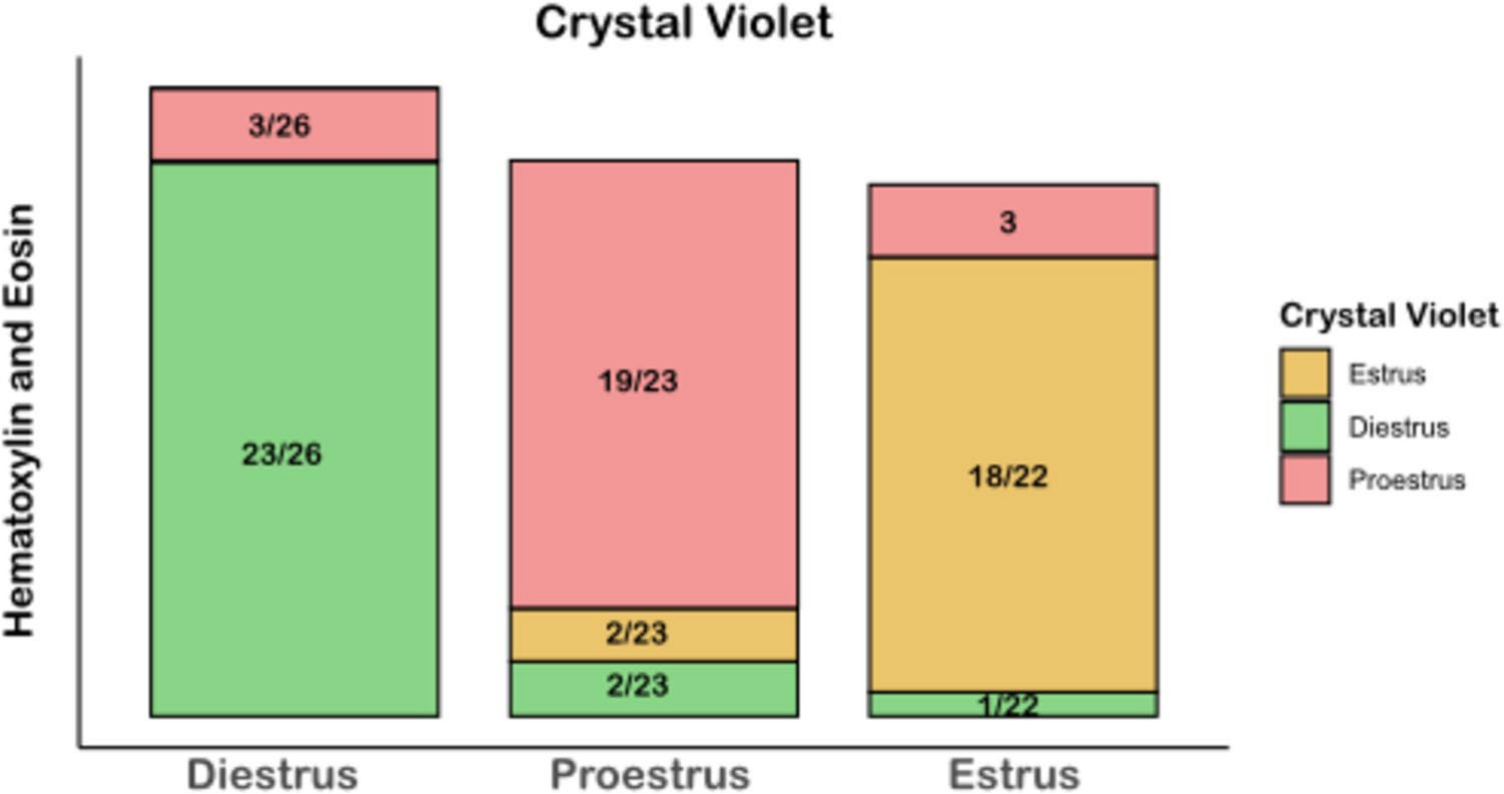
The agreement between crystal violet and hematoxylin–eosin in determining the phase of the estrous cycle. Cohen’s Kappa (κ) was used to assess the agreement between two methods, H&E and crystal violet, in determining the cycle phases in female rats. The calculated value of Cohen’s Kappa (κ = 0.76) indicates substantial agreement between the two methods

Baseline characteristics (heart rate, temperature, saturation, CO_2_) were not significantly different between the studied groups (Table 1). A substantial correlation (kappa 0.76) was observed between the crystal violet and hematoxylin–eosin staining methods used to identify the phase of the estrous cycle (Fig. 2).

**Table 1:** Baseline characteristics per group

Variable	Proestrus NPC	Estrus NPC	Diestrus NPC	Proestrus IPC	Estrus IPC	Diestrus IPC	Male NPC	Male IPC	Female OVX NPC	Female OVX IPC	Male ORX NPC	Male ORX IPC
N	12	11	15	11	11	11	11	11	13	14	11	13
Age (weeks)	8.3 ± 0.12	8.1 ± 0.6	8.1 ± 0.8	8.17 ± 0.4	8.3 ± 0.6	8.3 ± 0.6	8.2 ± 0.3	8 ± 0.6	10,2 ± 0.2	10,26 ± 0.2	10,6 ± 0.2	10,34 ± 0.2
Weight (g)	271 ± 11.2	273 ± 16.3	269 ± 19.3	286 ± 19.6	283 ± 23.9	288 ± 17.6	325 ± 28	337 ± 31.2	340.2 ± 31.2	334.2 ± 41.15	388.8 ± 13.9	388.2 ± 12.16
Temperature (°C)	37.7 ± 0.1	37.4 ± 0.17	37.4 ± 0.1	37.3 ± 0.11	37.3 ± 0.2	37.3 ± 0.11	37.6 ± 0.1	37.3 ± 0.1	37.3 ± 0.1	37.3 ± 0.1	37.4 ± 0.1	37.3 ± 0.1
Heart rate (BPM)	248 ± 33.5	246 ± 23.5	249 ± 23.5	222 ± 26.9	231 ± 28.3	229 ± 21.5	250 ± 18	244 ± 13	235 ± 22.9	230 ± 12.7	238 ± 22.9	242 ± 24.8
ET-CO_2_ (%)	5.0 ± 0.6	5.0 ± 0.6	5.1 ± 0.4	4.9 ± 0.5	4.9 ± 0.4	4.9 ± 0.5	5.9 ± 0.3	5.7 ± 0.7	5.9 ± 1	5.9 ± 1	5.5 ± 1	5.9 ± 1
O_2_ saturation (%)	98.9 ± 0.3	98.9 ± 0.3	98.9 ± 0.3	98.8 ± 0.2	98.9 ± 0.3	98.2 ± 0.2	99.0 ± 0.2	98.9 ± 0.3	98.2 ± 0.2	98.2 ± 0.2	98.2 ± 0.2	98.2 ± 0.2

Data are shown as mean and standard deviation

*IPC* ischemic preconditioning, *NPC* no ischemic preconditioning, *ET* end-tidal, *BPM* beats per minute, *ORX* orchiectomized male rats, *OVX* ovariectomized female rats

### Levels of sex hormones in female rats

**Fig. 3:**
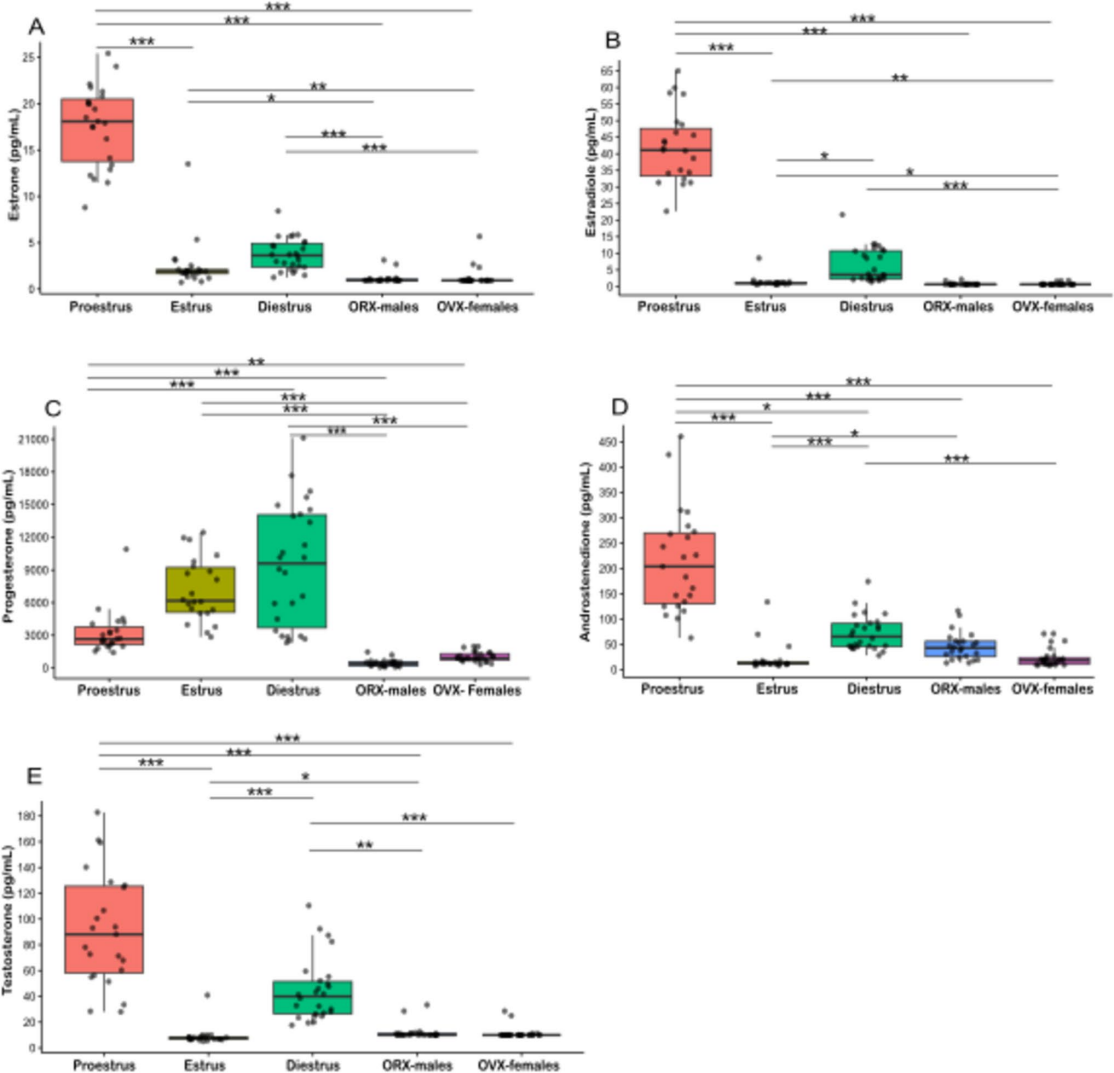
The levels of sex steroid hormones different to estrous stages in female rats and gonadectomized groups. Cyclical changes in plasma levels of estrone (**A**), estradiol (**B**), progesterone (**C**), androstenedione (**D**), and testosterone (**E**) were analyzed by gas chromatography-tandem mass spectrometry. Estrous cycle phase was determined by the predominant cell types in vaginal smears via microscopy. Data is presented as median and ranges. Statistical analysis was performed using the Independent-Samples Kruskal–Wallis Test to assess overall group differences, followed by Dunn’s Post-Hoc Test for pairwise comparisons. *p < 0.05, **p < 0.01, *** p ≤ 0.001

In the proestrus (P) phase, the endogenous levels of estradiol, estrone, androstenedione, and testosterone were significantly higher while progesterone was significantly lower compared to the estrus phase. In comparison to diestrus, proestrus showed significantly lower progesterone levels and significantly higher testosterone and androstenedione levels. In addition, in proestrus phase estradiol levels showed a trend toward significance compared to diestrus (*p* = 0.06). Compared to OVX-females and ORX-males, all hormone levels were significantly higher in the P phase. (Fig. 3 and Table 2).

**Table 2:** Plasma sex steroid hormones according to estrous phase in female rats

Hormones	Estrous phase	Median	Interquartile ranges
Estradiol	Proestrus	41.1	33.3–47.6
	Estrus	1.04	.847–1.11
	Diestrus	3.54	2.19–10.8
	OVX	0.625	0.625–0.625
	ORX	0.625	0.625–0.694
Androstendione	Proestrus	204.0	130–270
	Estrus	12.2	10.5–15.1
	Diestrus	65.3	45.7–91.2
	OVX	17.9	10.4–23.7
	ORX	42.8	25.7–56.0
Progesteron	Proestrus	2655.3	2156–3756
	Estrus	6174	5116–9234
	Diestrus	9610.7	3593–14,053
	OVX	923	731–1312
	ORX	391	187–571
Estrone	Proestrus	18.1	13.8–20.5
	Estrus	1.9	1.67–2.09
	Diestrus	3.6	2.39–4.93
	OVX	0.938	0.938–0.938
	ORX	0.938	0.938–1.04
Testosterone	Proestrus	88.11	58.3–125
	Estrus	7.63	6.6–8.45
	Diestrus	39.92	26.5–51.5
	OVX	10	10–10
	ORX	10	10–11.1

*ORX* orchiectomized male rats, *OVX* ovariectomized female rats

### Infarct size according to estrous cycle phase and ischemic preconditioning

**Fig. 4:**
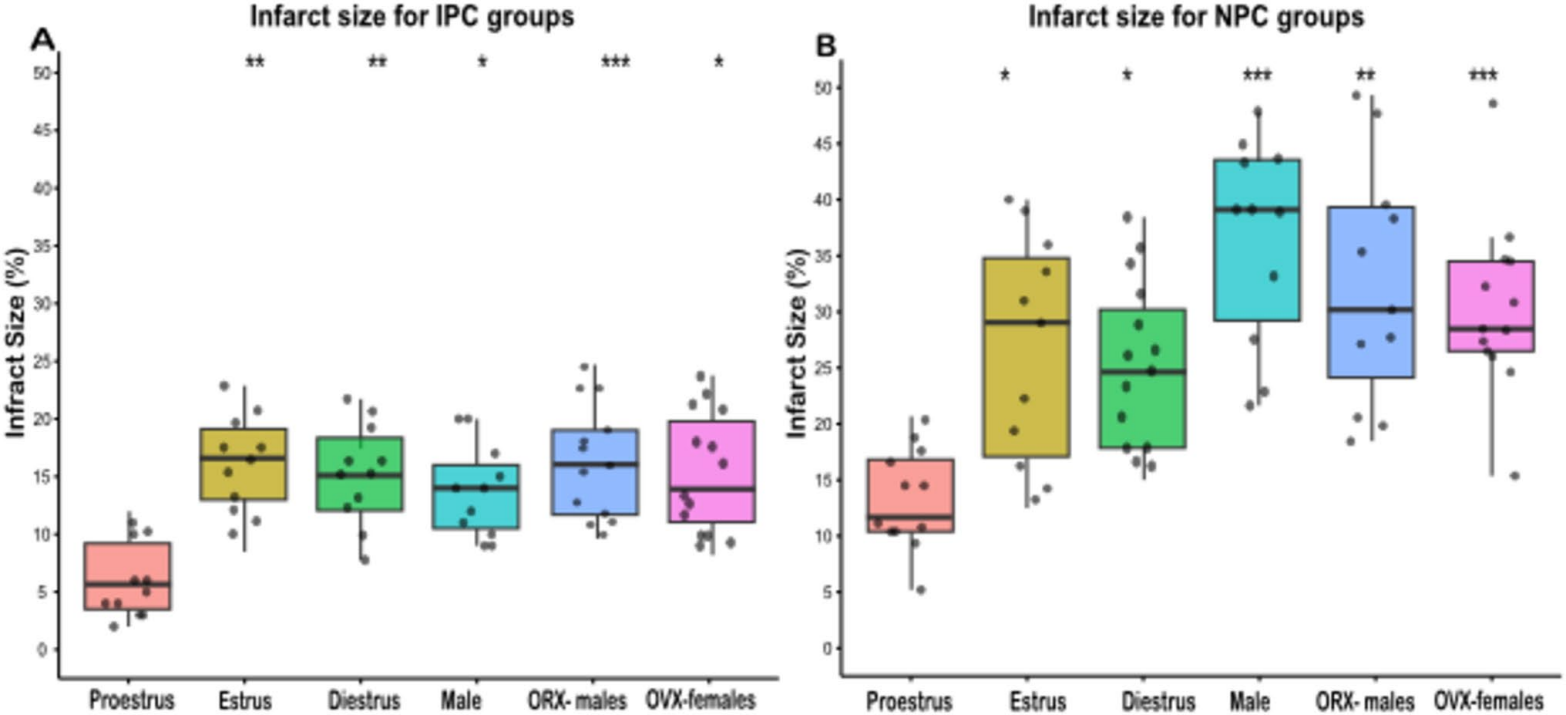
The infarct size in female rats across the estrous cycle phases, in male rats, and in gonadectomized groups. Infarct size, as measured via Evan’s blue/triphenyltetrazolium chloride double staining, is expressed as median and ranges. **(A)** infarct size for the six IPC groups. **(B)** infarct size for the six NPC groups. Infarct size was significantly smaller with IPC versus NPC in all groups (Proestrus, p = 0.019; Estrous, p = 0.035; Diestrus, p < 0.01; Male, p < 0.001; OVX, p < 0.001; ORX, p < 0.001). Statistical analysis was performed using the Independent-Samples Kruskal–Wallis Test to assess overall group differences, followed by Dunn’s Post-Hoc Test for pairwise comparisons. *p < 0.05, **p < 0.01. NPC = no ischemic preconditioning; IPC = ischemic preconditioning; ORX = orchiectomized rats; OVX = ovariectomized rats

In rats exposed to IPC, infarct size was significantly smaller across all groups compared to their respective non-preconditioned (NPC) groups. Myocardial infarct size was significantly smaller in females during the proestrus phase compared to females in other cycle phases, males, and gonadectomized rats. No significant differences were observed in pairwise comparisons of infarct size between estrus, diestrus, OVX-females, and ORX-males (Fig. 4A and Table 3).

**Table 3:** Myocardial infarct size according to estrous phase in female rats

Groups	Infarct size (%) Median	Interquartile ranges
Proestrus NPC	10.3	8.5–17
Estrus NPC	29.8	17–36.2
Diestrus NPC	25.7	16.4–33.2
Proestrus IPC	5.8	4.7–9.1
Estrus IPC	17.1	11.8–19.6
Diestrus IPC	15.7	12–19
Male NPC	38.4	28.5–43.3
Male IPC	14	10.5–16
Male ORX NPC	30	24.4–41.5
Male ORX IPC	15.2	12.8–17.5
Female OVX NPC	28.5	26.5–34.5
Female OVX IPC	12.5	9.88–18

Data are shown as median, and interquartile ranges

*NPC* no ischemic preconditioning, *IPC* ischemic preconditioning, *ORX* orchiectomized male rats; *OVX* ovariectomized female rats

Among rats not exposed to IPC, myocardial infarct size was significantly smaller in females during the proestrus phase compared to females in other cycle phases, males, OVX-females, and ORX-males. Pairwise comparisons in the NPC subset followed a pattern similar to that observed in the IPC subsets (Fig. 4B and Table 3).

### Multivariable linear regression models

The multivariable linear regression model examining the effects of IPC and sex revealed a statistically significant increase in infarct size in males (*p* < 0.001) compared to females (Table 4). In addition, a significant interaction was observed between sex and IPC, such that the effect of IPC on infarct size was greater in male rats.

**Table 4:** Multivariable linear regression model showing the effects of sex, preconditioning, and their interaction on myocardial infarct size

Variable	Estimate	95% CI	*p* value
Intercept	21.54	18.85–24.23	< 0.001
Sex			
Female	Reference		
Male	14.29	8.61–19.96	< 0.001
Ischemic preconditioning (IPC)			
NPC	Reference		
IPC	− 8.73	− 12.67–− 4.79	< 0.001
Interaction term			
Male: IPC	− 13.37	− 21.46–− 5.28	0.002

*NPC* no ischemic preconditioning, *IPC* ischemic preconditioning

**Fig. 5:**
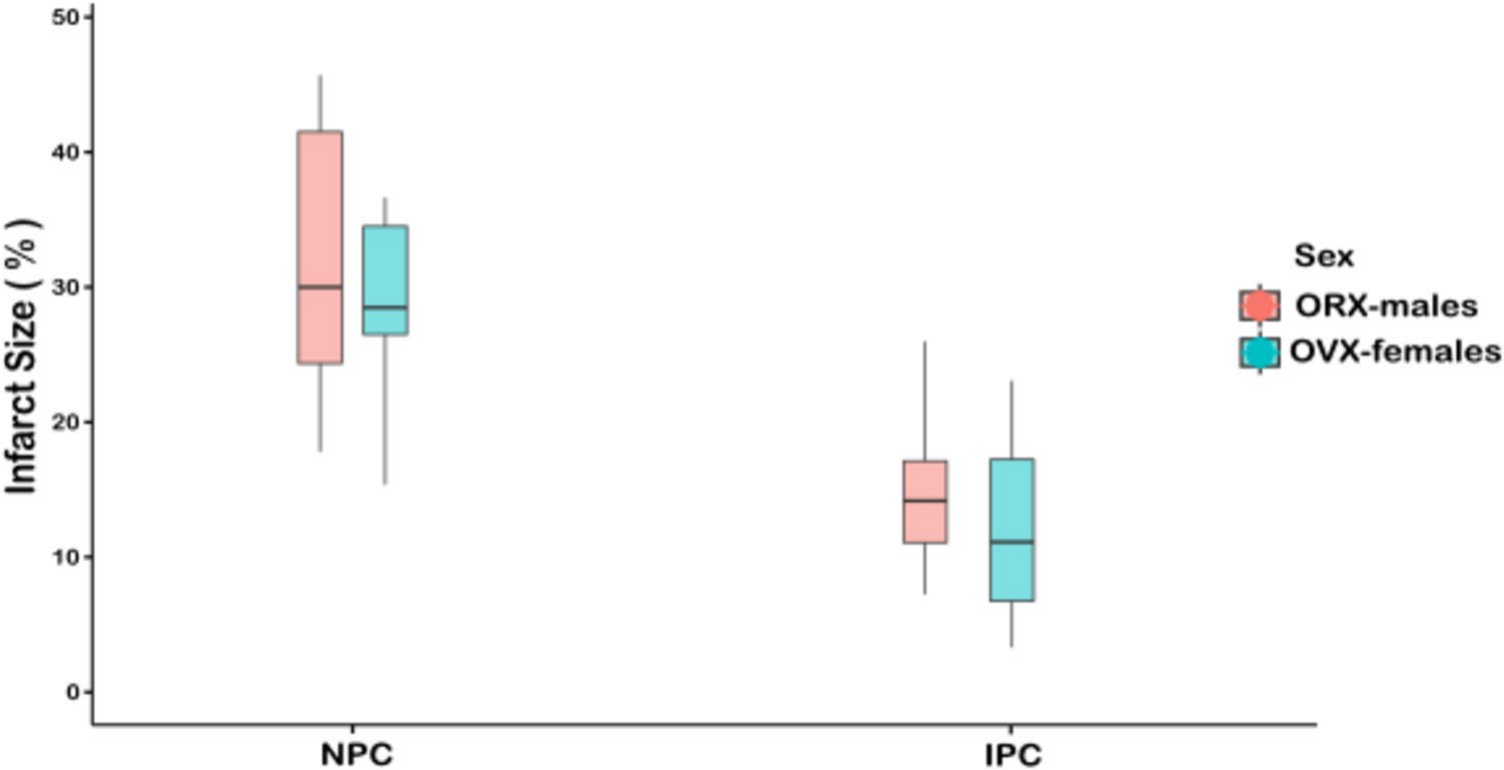
Infarct size predictions from multivariable linear regression models. Interaction between IPC and ovariectomized/orchiectomized groups. IPC = ischemic preconditioning; NPC, = no preconditioning; ORX = orchiectomized males; OVX = ovariectomized females

Among castrated rats, there was no significant association between sex and infarct size and no significant interaction between sex and the effect of IPC on infarct size, with IPC conferring similar protection in both sexes (Fig. 5, Table 5). When the M, F, OVX and ORX groups were evaluated in the same multivariable model in which F was the reference group, M, OVX, and ORX were all significantly associated with larger infarct size, and significant interactions were observed between IPC and M, OVX, and ORX (Table 6).

**Table 5:** Multivariable linear regression model showing the effects of sex and preconditioning, and their interaction on infarct size in gonadectomized rats

Variable	Estimate	95% CI	*p* value
Intercept	32.4	28.19–36.65	< 0.001
Gonadectomy			
ORX-males	Reference		
OVX-females	− 2.1	− 7.84–3.64	0.472
Ischemic preconditioning (IPC)			
NPC	Reference		
IPC	− 16.9	− 22.64–− 11.13	< 0.001
Interaction terms			
OVX-females: IPC	0.45	− 7.42–8.33	0.89

*NPC* no ischemic preconditioning, *IPC* ischemic preconditioning, *ORX-males* orchiectomized males, *OVX-females* ovariectomized females

**Table 6:** Multivariable linear regression model showing the effects of sex, gonadectomy, preconditioning, and their interaction on myocardial infarct size

Variable	Estimate	95% CI	*p* value
Intercept	21.5	18.97–24.12	< 0.001
Sex			
Female	Reference		
Male	14.29	8.85–19.72	< 0.001
ORX-males: IPC	10.87	5.44–16.31	< 0.001
OVX- females: IPC	8.77	3.67–13.87	< 0.001
Ischemic preconditioning (IPC)			
NPC	Reference		
IPC	− 14.182	− 18.26–− 10.10	< 0.001
Interaction term			
Male: IPC	− 13.373	− 21.12–− 5.62	< 0.001
ORX-males: IPC	− 8.16	− 15.68–− 0.64	0.03
OVX-females: IPC	− 7.709	− 14.89–− 0.52	0.03

*NPC* no ischemic preconditioning, *IPC* ischemic preconditioning, *ORX-males* orchiectomized males, *OVX-females* ovariectomized females

When female rats in the F group were grouped according to estrous cycle phases, with P as the reference group, both the other phases (E and D), as well as M, OVX, and ORX were all associated with larger infarct size. As in the previous model in which F rats were considered as a single group, there were significant interactions between M, OVX, and ORX and the effect of IPC on infarct size, while there was no statistically significant interactions between E or D and IPC (Table 7).

**Table 7:** Multivariable linear regression model showing the effects of cycle phase, preconditioning, and their interaction on infarct size

Variable	Estimate	95% CI	*p* value
Intercept	12.14	8.10–16.17	< 0.001
Estrous cycle / sex			
Proestrus	Reference		
Estrus	14.27	8.44–20.11	< 0.001
Diestrus	13.35	7.94–18.76	< 0.001
Male	23.69	17.86–29.52	< 0.001
ORX- males	20.28	14.44–26.11	< 0.001
OVX-females	18.18	12.58–23.77	< 0.001
Ischemic preconditioning (IPC)			
NPC	Reference		
IPC	− 5.37	− 11.211–− 0.455	0.04
Interaction terms			
Estrus: IPC	− 4.922	− 13.26–3.41	0.2437
Diestrus: IPC	− 4.570	− 12.61–3.47	0.2621
Male: IPC	− 16.731	− 25.07–− 8.39	< 0.001
ORX-males: IPC	− 11.523	− 19.69–− 3.35	< 0.001
OVX-females: IPC	− 11.066	− 19–− 3.13	< 0.001

*NPC* no ischemic preconditioning, *IPC* ischemic preconditioning, *ORX-males* orchiectomized males, *OVX-females* ovariectomized females

In analyses restricted to the F group that assessed the effect of sex hormones on infarct size, estradiol was the only hormone significantly associated with a reduction in infarct size (*p* < 0.001, Table 8).

**Table 8:** Multivariable linear regression model showing the effects of sex hormones, preconditioning, and their interaction on infarct size

Variable	Estimate	95% CI	*p* value
Intercept	35.78	26.67–44.90	< 0.001
Testosterone	0.08	− 0.86–1.03	0.86
Progesterone	− 0.51	− 1.61–0.57	0.35
Estradiol	− 4.29	− 5.86–− 2.73	< 0.001
IPC	− 15.44	− 19.48–− 11.41	< 0.001
Interaction term			
Estradiol: IPC	1.96	− 0.19–4.11	0.07

*NPC* no ischemic preconditioning, *IPC* ischemic preconditioning

Across all models, ischemic preconditioning consistently showed an association with smaller infarct sizes (p < 0.05 for all).

## Discussion

The main findings of our study include: (i) Female rats in the proestrus phase show smaller infarct size following myocardial ischemia–reperfusion compared to female rats in other phases of the estrous cycle, male rats, and gonadectomized rats. (ii) Levels of estradiol and estrone are inversely correlated with infarct size, suggesting these hormones could mitigate the severity of myocardial infarction after ischemia–reperfusion injury. (iii) The infarct size-reducing effects of ischemic preconditioning were more pronounced in males than females. However, this difference was no longer observed after gonadectomy, with IPC showing similar efficacy in ORX-males and OVX-females. These findings suggest that some of the protective mechanisms typically induced by IPC may already be active in females during the proestrus phase, likely mediated by higher levels of estradiol.

Sex-based differences in prognosis after myocardial infarction are well-documented. Understanding and addressing these differences is critical to ensuring that research findings are accurately interpreted and applicable across populations [4]. A central point of debate in this context is the role of sex hormones, particularly estrogen, in influencing myocardial infarction outcomes.

Estrogens has been proposed as being a primary candidate to exert cardioprotective effects [9, 26, 29]. However, their clinical significance remains controversial. While some clinical trials have shown that therapies based on estrogen, either alone or combined with progesterone, fail to significantly affect the prognosis for myocardial infarctions in both pre- and postmenopausal women, other research suggests that the timely administration of estrogen may mitigate ischemic injury [9, 17, 41].

Our study showed that the infarct size in rats during the proestrus phase, which corresponds to the follicular phase in women and is characterized by high-estradiol and estrone levels, was significantly smaller compared to the diestrus and estrus phases, males, and gonadectomized rats. In addition, our study indicates that estradiol and estrone were associated with a smaller infarct size. These findings align with other studies reporting cardioprotective effects of estrogen [9, 35]. Several biological mechanisms may underlie the beneficial effects of estrogens on cardiovascular diseases in women. Nitric oxide synthase (NOS) isoforms have been shown in many tissues to increase in response to estrogen. Estrogen results in upregulation of cardiac eNOS and nNOS which have been shown previously to be important mediators of cardioprotection [28]. Estrogens can promote vasodilation by increasing plasma concentrations of endothelium-derived relaxing factor nitric oxide, and can also inhibit the renin–angiotensin system by reducing the transcription of angiotensin-converting enzyme, regulating specific inflammatory markers and cytokines [41]. Moreover, numerous studies indicates that estradiol has preconditioning effects on the heart [40] similar to IPC, which is known to protect the heart against ischemia/reperfusion injury [8]. These protective effects could be a reason for the different infarct size and differentiated efficacy of IPC across the different cycle phases. In previous studies, these effects are thought to be mediated by the activation of cardiomyocyte K_ATP_ channels, activation of the PI3-K/PKB pathway, involvement of members of the PKC family of protein kinases, and elevation of intracellular ROS levels [8, 40] This suggests that estradiol may confer a protective effect by mediating a set of universal signaling events shared with IPC.

Findings from the multivariable linear regression analyses showed that males and gonadectomized groups developed larger infarcts compared to females overall, with the smallest infarcts observed in females during the proestrus phase. Conversely, the protective effects of IPC were more pronounced in males and gonadectomized groups than in females, with the least pronounced effects in females during the proestrus phase. In addition, the analysis of sex hormones identified estradiol as the only hormone significantly associated with infarct size reduction, with a trend toward significance in its interaction with IPC.

A possible explanation unifying these observations is that IPC and estrogen exert their protective effects through partially overlapping pathways. Our findings support the hypothesis that IPC and estrogen share common cardioprotective pathways, with a less pronounced effect of IPC when these cardioprotective pathways are partially activated by estrogen [40].

The estrous cycle of the rat is short and characterized by varying levels of sex hormones, which can introduce variability in experimental outcomes. This variability may explain the greater scatter in infarct size observed in studies that include female animals in cardioprotection experiments [7, 20, 23]. Despite this, previous studies comparing sex differences have frequently neglected to account for the estrous cycle phase in the study design [20, 23]. Consequently, it is possible that a significant portion of the subjects in previous studies were in different estrous cycle phases, impacting the homogeneity of the groups and possibly explaining the lack of observed differences. In our study, we accounted for the estrous cycle phases by grouping female rats accordingly and confirming these phases through hormone level measurements and vaginal smear analysis. This approach ensures the accuracy of phase identification and enhances the homogeneity of the groups [14, 32].

As reported in the literature, a previous study reported no correlation between serum levels of 17β-estradiol and myocardial infarct size during the estrous cycle [13], a finding that contradicts our results. One potential reason for this distinction is that in their study, estradiol levels were assessed using ELISA, which may have methodological challenges associated with detection limits. The characterization of the sex steroids in rat models requires precise and sensitive methods, taking into account the limited plasma volumes available from rodents. In contrast, our study used gas chromatography-tandem mass spectrometry for sex hormone evaluation, ensuring the acquisition of accurate results. GC–MS/MS is a highly sensitive and specific method that allows the analysis of sex steroid concentrations in samples with low hormone levels [32]. In addition, the representative images for each cycle phase as presented in the conflicting study deviated significantly from the standard cycle phase images in existing literature. In contrast, to ensure precise cycle phase identification, our study combined both microscopic analysis of vaginal smears and hormone level confirmations, resulting in enhanced accuracy. These methodological differences likely contributed to the divergence in findings between the studies.

## Conclusion

In female rats subjected to myocardial ischemia–reperfusion, infarct size was significantly smaller during the proestrus phase compared to other phases and compared to males; a difference that appeared to be mediated by estrogens. Conversely, the protective effects of IPC on reducing infarct size were less pronounced in females in the proestrus phase than in males; suggesting that the mechanisms behind estrogen-induced and IPC-induced cardioprotection may be partially overlapping. These findings underscore the importance of accounting for estrous cycle stages in experimental designs involving female animals, and the possibility that sex hormones may affect the efficacy of cardioprotective strategies.

## Limitations

We did not investigate the potential influence of other reproductive hormones (prolactin, follicle-stimulating hormone, luteinizing hormone) involved in the estrous cycle on ischemia/reperfusion injury. Furthermore, the duration of ischemia and reperfusion protocols used in our study may differ from the clinical scenario, limiting the direct translation of our findings to human patients with myocardial infarction.

## Data Availability

The raw data behind the conclusions of this study are available from the corresponding author upon reasonable request.

## Abbreviations

CHD: Coronary heart disease
GC**–**MS/MS: Gas chromatography-tandem mass spectrometry
H&E: Hematoxylin–eosin
IPC: Ischemic preconditioning
LAD: Left anterior descending artery
NPC: No ischemic preconditioning
OVX: Ovariectomized
ORX: Orchiectomized
TTC: Triphenyltetrazolium chloride
